# Blood Clotting Factor VIII: From Evolution to Therapy

**Published:** 2013

**Authors:** N. A. Orlova, S. V. Kovnir, I. I. Vorobiev, A. G. Gabibov, A. I. Vorobiev

**Affiliations:** 1Center “Bioengineering”, Russian Academy of Sciences, 60-letija Oktyabrja av., 7/1, Moscow, Russia, 117312; 2Shemyakin and Ovchinnikov Institute of Bioorganic Chemistry, Russian Academy of Sciences, Miklukho-Maklaya Str., 16/10, Moscow, Russia, 117997; 3Research Center for Hematology, Ministry of Health and Social Development of the Russian Federation, Novij Zykovsky proezd, 4, Moscow, Russia, 125167

**Keywords:** blood clotting factor VIII, hemophilia A, heterologous protein expression systems

## Abstract

Recombinant blood clotting factor VIII is one of the most complex proteins for industrial manufacturing due to the low efficiency of its gene transcription, massive intracellular loss of its proprotein during post-translational processing, and the instability of the secreted protein. Improvement in hemophilia A therapy requires a steady increase in the production of factor VIII drugs despite tightening standards of product quality and viral safety. More efficient systems for heterologous expression of factor VIII can be created on the basis of the discovered properties of its gene transcription, post-translational processing, and behavior in the bloodstream. The present review describes the deletion variants of factor VIII protein with increased secretion efficiency and the prospects for the pharmaceutical development of longer acting variants and derivatives of factor VIII.

## INTRODUCTION

Blood clotting factor VIII (FVIII) is the nonenzymatic cofactor to the activated clotting factor IX (FIXa), which, when proteolytically activated, interacts with FIXa to form a tight noncovalent complex that binds to and activates factor X (FX). *FVIII *gene defects may cause hemophilia A, the X-linked recessive genetic disorder with an incidence rate of ~ 1 case per 5,000 males. Approximately half of all hemophilia A cases are caused by inversions in intron 22 of the *FVIII *gene [1]; an additional 5% are caused by intron 1 inversions. By November 2012, a total of 2,107 various mutations in the *FVIII *gene with the hemophilia A phenotype had been described in the HAMSTeRS (The Hemophilia A Mutation, Structure, Test and Resource Site) database [2]. By July 2012, a total of 2,537 such mutations had been listed in the CHAMP (The CDC Hemophilia A Mutation Project) database [3].

Continuous substitution therapy using FVIII drugs is the only efficient treatment for hemophilia A. The conventional source of FVIII is donated blood plasma, its supply being limited. Even after a thorough screening of the prepared plasma units and numerous procedures of viral inactivation, the risk of transmission of viral [4, 5] and prion infections [6] remains when plasma is used as a source for production of therapeutic proteins. Recombinant human factor VIII for hemophilia A treatment can be produced using cultured mammalian cells or the milk from transgenic animals.

## FUNCTIONS OF FACTOR VIII IN THE HEMOSTATIC SYSTEM

**Fig. 1:**
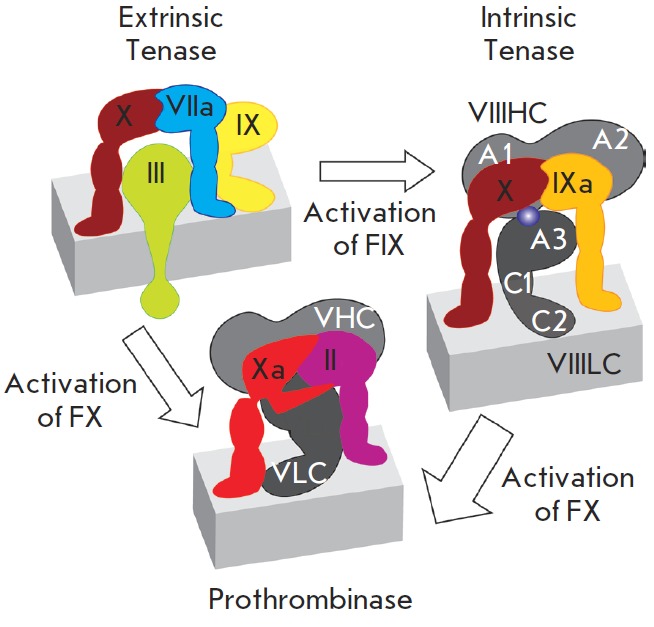
Tenase complex assembly on the cell membrane. Intact proenzymes of coagulation factors are denoted by Roman numerals, activated enzymatic factors are denoted by the letter “a”. Blood clotting factors are bound to the membrane surface, Factor III is the integral membrane protein. VIIIHC – heavy chain of Factor VIII, VIIILC – light chain of Factor VIII, domains A1, A2, A3, C1, C2 of Factor VIII are denoted by white letters. VHC – heavy chain of Factor V, VLC – light chain of Factor V. The thickness of the reaction arrows corresponds to the reaction rates

The tight noncovalent FVIIIa–FIXa complex is formed on the phospholipid membrane surface and additionally binds the FX molecule, which is subsequently activated by FIXa. The activated FX leaves the complex and, in turn, triggers the conversion of prothrombin to thrombin (FII to FIIa), which directly converts fibrinogen to fibrin, the major component of blood clots (* Fig. 1 *). The ternary complex of clotting factors FIXа, FVIIIa, and FX, bound to the phospholipid membrane, usually is referred to as X-ase or tenase, and is the main element of the positive feedback loop in the blood clotting cascade. A complex that is functionally similar to tenase can be described for the extrinsic clotting pathway (FIII, FVIIa, FIX, FX); however, its enzymatic efficiency is considerably lower than that of “intrinsic” tenase. The unique feature of the tenase complex is the high degree of enhancing the catalytic activity (by approximately five orders of magnitude) of low-activity proteinase FIXa by the FVIIIa [7]. This enhancement occurs due to the changes in the active site conformation in FIXa as it binds to FVIIIa [8]. Factor V, homologous to factor VIII, potentiates the activity of FXa within the prothrombinase complex with the coefficient of enhancement of catalytic activity X240.

The functional activity of FVIII is measured *in vitro* by determination of the clotting time of the blood plasma sample with depleted endogenous FVIII and added FVIII in the solution under study. FVIII stability in the bloodstream was studied in model animals with a defective or deleted *FVIII *gene. The animal models of hemophilia A were discussed in review [9].

## STRUCTURE OF THE FVIII GENE AND ITS EXPRESSION FEATURES

**Fig. 2:**
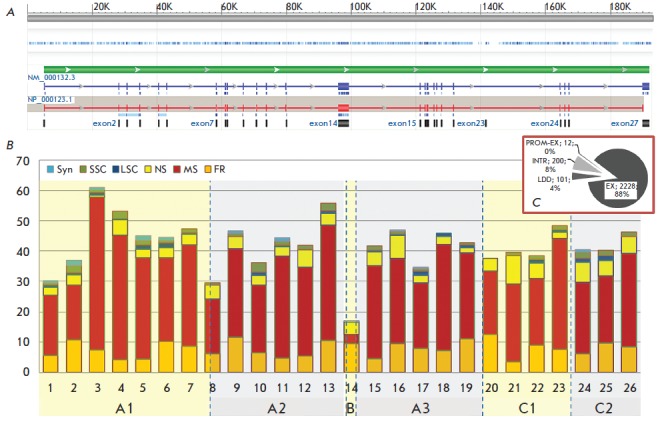
FVIII gene structure and frequencies of the mutations causing hemophilia A. Panel A: FVIII gene on the X chromosome, NCBI reference sequence number: NG_011403.1. Transcribed are two products of alternative splicing. Functional protein FVIII is coded by the transcription variant 1, reference number of mRNA NM_000132.3, reference number of the protein NP_000123.1. Panel B: Variants of mutations in FVIII gene exons according to [3]. The number of different recorded mutations per 100 bp of the coding sequence is shown. Abbreviations: NS – nonsense mutation; MS – missense; FR – frameshift; SSC – small structural change (in-frame, < 50 bp=""); LSC="" –="" large="" structural="" change="" (>50 bp). Numbers of histogram columns correspond to the exon numbers, names of protein domains are stated below the exon numbers. Length of exon 1 in mRNA is 314 b; the only coding part of this exon (including the signal peptide), 143 b, was included in the calculations; length of exon 26 in mRNA is 1965 b, the only coding part of this exon – 156 b – was used in the calculations. The lengths of the other exons are as follows: 2 – 122 b, 3 – 123 b, 4 – 213 b, 5 – 69 b, 6 – 117 b, 7 – 222 b, 8 – 262 b, 9 – 172 b, 10 – 94 b, 11 –215 b, 12 – 151 b, 13 – 210 b, 14 – 3106 b, 15 – 154 b, 16 – 213 b, 17 – 229 b, 18 – 183 b, 19 – 117 b, 20 – 72 b, 21 – 86 b, 22 – 156 b, 23 – 145 b, 24 – 149 b, 25 – 177 b. Panel C: Variants of mutations in the FVIII gene. Abbreviations: LDD – large deletions and duplications in one or multiple domains of FVIII; INTR – distortions in the splice sites; PROM-EX – promoter area mutations and deletions in the promoter area plus the exon; EX – mutations in the exons. Primary data taken from [3], extracted July 18, 2012

The *FVIII *gene localized on the long arm of the X chromosome occupies a region approximately 186 kbp long and consists of 26 exons (69–3,106 bp) and introns (from 207 bp to 32.4 kbp). The total length of the coding sequence of this gene is 9 kbp [10, 11] (*Fig. 2*). Expression of the *FVIII *gene is tissue-specific and is mostly observed in liver cells [12–14]. The highest level of the mRN A and FVIII proteins has been detected in liver sinusoidal cells [15, 16]; significant amounts of FVIII are also present in hepatocytes and in Kupffer cells (resident macrophages of liver sinusoids).

## DOMAIN STRUCTURE

The mature factor VIII polypeptide consists of 2,332 amino acid residues (the maximum length) and includes the A1–A2–B–A3–C1-C2 structural domains [17, 18] (*Fig. 2*). Three acidic subdomains, which are denoted as a1–a3 – A1(a1)–A2(a2)–B–(a3)A3–C1–C2, localize at the boundaries of A domains and play a significant role in the interaction between FVIII and other proteins (in particular, with thrombin). Mutations in these subdomains reduce the level of factor VIII activation by thrombin [19, 20]. There currently is some controversy regarding the accurate definition of the boundaries of FVIII domains; the most common versions are listed in*Table 1*.

**Table 1:** Domain architecture of factor VIII with indication of the domain borders

A1	a1	A2	a2	B	a3	A3	C1	C2	Reference
1–329	331-372	380-711	700-740	741-1648	1649-1690	1649-2019	2020-2172	2173-2332	[21]
1–336	337-372	372-710	711-740	741-1648	1649-1689	1649-2019	2020-2172	2173-2332	[22]
1–336		372-710		741-1648		1649-2019	2020-2172	2173-2332	[23]
1–336	337-374	375-719	720-740		1649-1689	1691-2025			[24]

**Fig. 3:**
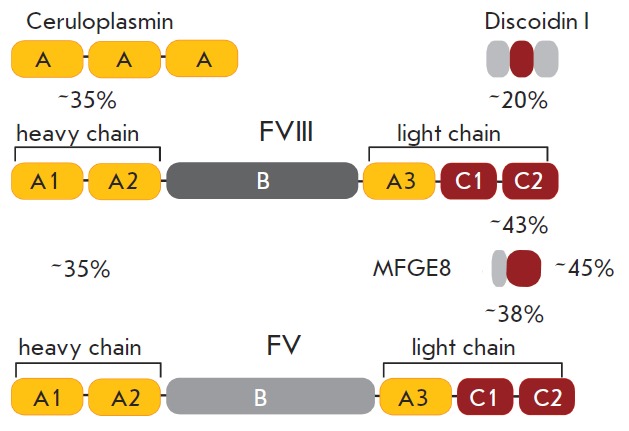
Domain structure of FVIII homologues. Numbers represent the homology level of amino acids for domain groups. Discoidin I was obtained from D. discoideum; all other proteins were obtained from H. sapiens

**Fig. 4:**
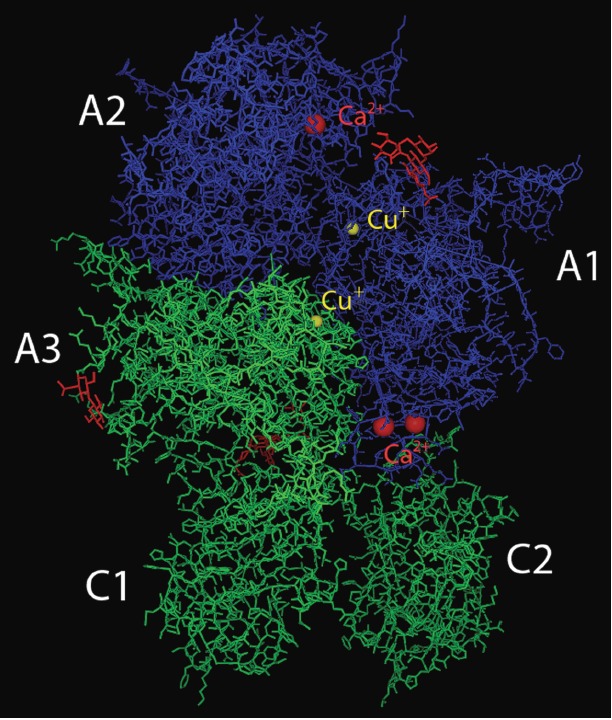
3D structure of the FVIII deletion variant according to [42]. Core residues of N-linked glycans are shown in red, FVIII heavy chain is shown in blue, light chain is shown in green

**Fig. 5:**
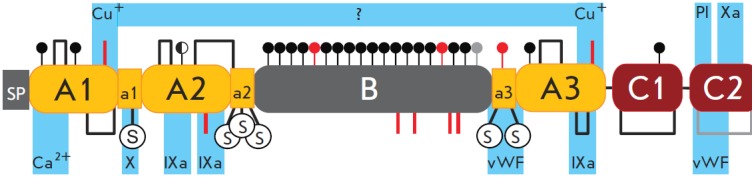
Post-translational modifications and functional sites of FVIII. N-glycosylation sites are denoted by circles. Filled black circles – occupied sites, half-filled circle – partially occupied site, filled gray circle – presumably occupied site, red circles – unoccupied sites. Disulphide bonds are denoted by brackets, gray bracket – presumably existing disulphide bond. Red vertical lines – reduced Cys residues, the actual state of Cys residues in the B domain is unknown. S inside a circle – sulfated Tyr residues. Light blue marks the areas of interaction with corresponding clotting factors, phospholipids (Pl), von Willebrand factor (vWF), and copper ions (Cu+). SP – signal peptide and propeptide

The FVIII A domains show 30% homology with each other, the A domains of factor V, and the copper-binding protein of human plasma, ceruloplasmin (* Fig. 3 *). The FVIII A1 domain coordinates a copper ion [17, 25–27] (*Fig. 4*). The region 558–565 of the A2 domain determines the binding of factor IXa and its conformational rearrangement within tenase [28] (*Fig. 5*).

The C1 and C2 domains in the light chain of mature FVIII are homologous to the C1 and C2 domains of FV [29], the C-terminal domains of the MFGE8 protein (milk fat globule-EGF factor 8, lactadherin) [30, 31], and the discoidin I fragment [32] (*Fig. 3*). These domains are capable of binding glycoconjugates and acidic phospholipids [33]. The C2 domain in FVIII is also required for binding to the von Willebrand factor (vWF) and ensuring selective interaction with phosphatidylserine in cell membranes [34] (*Fig. 5*).

(Fig.4 )

The B-domain encoded by a single long exon is partially removed from the mature protein. The B-domain contains 25 potential N-glycosylation sites, 16–19 of which are occupied and exhibit a significant level of microheterogeneity. The homology of FVIII B-domains in humans and mice is low; however, these domains are highly glycosylated in both species, which can attest to the significance of this modification for post-translational processing of a protein [35].

Proceeding from the significant homology of the factors V and VIII, a hypothesis has been put forward that the evolutionary origin of the *FVIII *gene is connected with duplication. Interestingly, the functional A and C domains of these proteins are conserved, while the similarity between the B-domains is limited to a high degree of glycosylation, which also attests to the functional significance of the high density of oligosaccharide groups in the FVIII B-domain [17, 25–27].

The highly glycosylated B-domain can participate in the intracellular transport of the FVIII precursor and its processing. However, abundant experimental data have demonstrated that deletion of the B-domain region enhances the secretion of functionally active FVIII [36, 37].

## COORDINATED METAL IONS

The interaction between the FVIII polypeptide chain and metal ions determines the structural integrity of the mature protein and its cofactor function. The presence of copper ions within FVIII has been demonstrated by atomic adsorption spectrometry; dissociation of the FVIII chains results in complete dissociation of copper ions [38]. In turn, the reassociation of the split FVIII chains is possible only in the presence of copper salts [39]. It has been established by electron paramagnetic resonance (EPR) that the coordinated copper ions within FVIII are reduced to the state of +1 (Cu^+^) [40]. The presence of two coordinated copper ions in direct contacts with the H267, C310, H315 and H1954, C2000, and H2005 residues (i.e., two valid type I binding sites of the copper ion) has been detected in crystals of the deletion FVIII variant (BDD SQ variant) [41] (*Fig. 4*). Both copper ion binding pockets localize near the contact surface of the A1 and A3 domains; however, they do not directly participate in the formation of noncovalent bonds between the domains. Simultaneously, evidence of functional significance has been obtained only for the binding site of copper ions in the A1 domain both via point substitution of cysteine residues [40] and by direct monitoring of the coordination of copper ions by FRET [39]. The C310F mutation in the *FVIII *gene [2] causing a severe form of hemophilia A is an additional argument in favor of the physiological significance of the copper-binding site in the A1 domain.

Both copper ions and calcium or manganese ions are required to recover the procoagulant activity of FVIII during chain dissociation–re-association [43, 44]. Calcium or manganese ions do not affect chain dimerization, but they ensure that the heterodimer FVIII molecule has an active conformation [39] as they bind to the sites located on both protein chains [45, 46]. The major Ca^2+^- binding site localizes in the A1 domain (region 108–124) [45] and is homologous to the corresponding site in the FV molecule [47]. It has been ascertained by alanine scanning that Ca^2+^ binding is mediated by the D116, E122, D125, and D126 residues, while the interaction with Mn^2+^ is mediated by the D116 and D125 residues [48].

## POST-TRANSLATIONAL PROCESSING OF THE FVIII PRECURSOR PROTEIN

FVIII is synthesized in the liver, which has been supported by the fact that liver transplantation can cure hemophilia A. When isolating and purifying liver cell populations, it has been ascertained that secretion of significant amounts of FVIII (0.07 IU/million cells/day) is observed in primary cultures of liver sinusoidal endothelial cells [15]. No successful attempts to immortalize cultured liver sinusoidal endothelial cells have been documented thus far; hence, all the experimental data on the features of FVIII biosynthesis have been obtained using heterologous expression systems that are usually characterized by artificially increased productivity [49].

Translocation of the growing FVIII polypeptide chain to the lumen of the endoplasmic reticulum (ER ), processing of the signal 19-amino-acid-long peptide, and the primary events of disulfide bond formation and attachment of the high-mannose nuclei of N-linked oligosaccharides to the FVIII chain seem not to limit the total rate of its biosynthesis and have been thoroughly described in [47]. Meanwhile, the subsequent events of modifying oligosaccharide chains, disulfide isomerization, and folding of FVIII molecules may overload the corresponding enzyme groups in the cell and activate the systems responsible for retention of the incorrectly processed proteins in ER or the recycling systems for these proteins. The total rate of FVIII secretion is believed to be limited by translocation of the FVIII precursor from the ER to the Golgi apparatus; the FVIII polypeptide can remain in the ER for 15 min to several days.

## N-GLYCOSYLATION

**Fig. 6:**
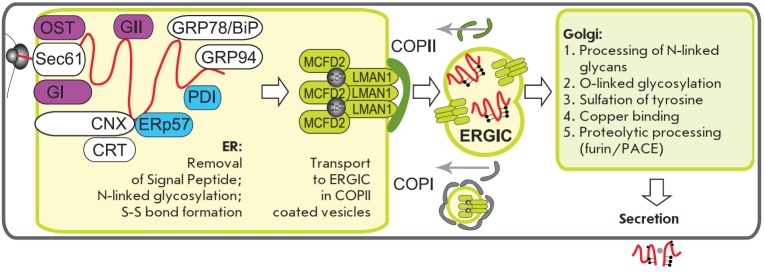
Intracellular traffic of the FVIII polypeptide to be secreted. OST – Oligosaccharyltransferase, Sec61 – membrane protein translocator, GI and GII – glucosidases I and II; CNX – calnexin, CRT – calreticulin, GRP78/BiP – glucose regulated protein 78 / immunoglobulin binding protein; GRP94 – glucose regulated protein 94; PDI, ERp57 – disulphide isomerases; MCFD2 – multiple coagulation factors deficiency protein 2; LMAN1 – mannose-binding lectin 1; COPI, COPII – vesicle coat proteins I and II; ERGIC – ER-Golgi intermediate compartment

After the primary N-glycosylation of the FVIII chain and cleavage of the two first glucose residues from oligosaccharide groups by glucosidases I and II (GTI, GTII), polypeptide FVIII binds to the lectins calnexin (CN X) and calreticulin (CRT ), which prevent the secretion of an immature protein [50] (*Fig. 6*). After the third glucose residue is eliminated, the protein is normally released from its complex with CN X and CRT and is transferred to the Golgi apparatus. Meanwhile, the unfolded or incorrectly folded FVIII remains in the ER , where it undergoes re-glucosylation by the UGT enzyme (UDP-glucose:glycoprotein glucosyltransferase) [51]. Next, it binds to CN X and CRT and undergoes shortening of GTII again (the so-called calnexin cycle).

The incorrectly folded FVIII molecules, along with the other proteins, are transferred from the ER to cytosolic proteasomes via the ER AD (ER -associated degradation) pathway; the elimination of the polypeptide from the calnexin cycle is mediated by the specialized EDEM protein [28]. Indeed, it has been demonstrated in pulse-chase experiments with proteasomes inactivated by lactacystin [50] that a significant portion of FVIII undergoes degradation via the ER AD pathway instead of being translocated to the Golgi apparatus; however, in those experiments proteasome inactivation increased the amount of intracellular FVIII but not its concentration in the culture medium. Thus, the ER AD pathway alone does not eliminate significant amounts of FVIII from the lumen of the ER and cannot be the reason for the limited transfer of FVIII from the ER to the Golgi apparatus. Since the major fraction of Nlinked oligosaccharides in the FVIII molecule is localized in the B-domain, the deletion FVIII variants are less susceptible to retention in the ER during the calnexin cycle, which partially explains their increased level of secretion.

## DISULFIDE BOND FORMATION

According to results of 3D modeling of FVIII and the results of most experimental studies, the FVIII molecule contains eight disulfide bonds: two in each A domain, one in each C domain, and three reduced Cys residues, one each in the A1, A2, and A3 domains (*Fig. 5*). There are no conclusive data on the state of the cysteine residues in the B-domain. Seven out of eight disulfide bonds localize within a polypeptide globule, while the C1899–С1903 bond (A3 domain) is exposed on the surface. When conducting a series of substitutions of cysteine residues by serine or glycine residues, S.W. Pipe *et al*. found that all the seven non-exposed disulfide bonds are required to maintain the structural integrity of the FVIII molecule, while the removal of the 1899–1903 bond improves secretion of FVIII twofolds without affecting its functional activity [52]. It is rather possible that the removal of the only exposed disulfide bond results in suppression of FVIII retention in the ER occurring due to the translocation control by the disulfide isomerases [53]. However, the specific mechanism of such control with respect to FVIII and the participating proteins has hardly been studied.

## FOLDING AND INTERACTION WITH ER CHAPERONES

Factor VIII in the lumen of the ER forms a strong complex with the major ER chaperone GRP78 (glucoseregulated protein MW 78.000), which is also known as BiP (immunoglobulin-binding protein) [54] and is one of the key components of the UPR (unfolded protein response) signaling pathway. BiP synthesis is typically induced when cells experience glucose starvation, during N-glycosylation inhibition, and in the presence of incorrectly folded proteins in the ER [55] (in particular, during FVIII overexpression) [56]. It should be mentioned that overexpression of human FVIII in cultured cells results in total activation of UPR, which manifests itself not only as a positive regulation of BiP, but also as activation of the *ERSE *gene and an increase in the level of splicing of *XBP1 *mRN A [57]. Thus, there can be other chaperones (in addition to BiP) that initiate the activation of UPR when large amounts of FVIII are transported into the lumen of the ER.

The BiP–polypeptide complex exhibits ATPase activity; hydrolysis of ATP is required to ensure disintegration of the complex. The isolation of FVIII from BiP and secretion require exceptionally high ATP expenditures [58].

Unlike FVIII, its homologue factor V does not interact with BiP. The site of FVIII binding to BiP (a hydrophobic β-sheet within the A1 domain lying near the C310 residue, which is a component of the type I copper-ion binding site) was identified using a series of chimeric FVIII–FV proteins [40, 59]. BiP forms direct contacts with hydrophobic amino acids, and the point mutation F309S inside this β-sheet results in a threefold increase in FVIII secretion, which correlates with reduced ATP expenditure [59]. Since the F309 residue is adjacent to C310, the key residue in the copper-coordination site in the A1 domain, one can assume that BiP interacts in the attachment of copper ions to FVIII as well.

Approximately one-third of FVIII molecules in the ER are aggregated to noncovalent multimers. The replacement of the region 227–336 of FVIII by the homologous region of FV reduces its degree of aggregation and affinity to BiP, as well as increases secretion [60]. The functional value of the FVIII-BiP complex presumably consists in the retention of FVIII in the ER , rather than in ensuring efficient FVIII folding prior to its translocation to the Golgi apparatus.

## TRANSPORT OF FVIII FROM ER TO THE GOLGI APPARATUS

Transport of the FVIII polypeptide from the ER to the Golgi apparatus occurs via the ER -Golgi intermediate compartment (ER GIC) (*Fig. 6*). FVIII and FV are recruited to this compartment by binding to the transmembrane protein (cargo receptor) ER GIC-53, which is also known as LMAN1 (lectin, mannose-binding, 1) and ensures mannose-selective, calcium-dependent binding and transport of glycoproteins from the ER to the Golgi apparatus [61].

The mutations resulting in the loss of LMAN1 function or disturbing the interaction between LMAN1 and the component of the transport complex MCFD2 (multiple coagulation factor deficiency protein 2) cause inherited coagulopathy, a combined deficiency of factor V and factor VIII [62–64]. The FVIII level in the plasma of patients with mutant LMAN1 decreases to 5–30% of its normal level [65].

The transport of four proteins (FV, FVIII and the lysosomal proteins, the cathepsins catC and catZ) through the intermediate compartment with the participation of the LMAN1–MCFD2 has been confirmed by the cross-linking method [66, 67]. A number of other proteins interact with LMAN1, but not with MCFD2, as they are transported [68]. It has been detected by crosslinking that 5–20% of the total intracellular FVIII localizes in the complex with LMAN1 and MCFD2 [69]. Calcium ions are required for the FVIII complex with both partners to form; meanwhile, the FVIII–MCFD2 can be formed independently of LMAN1. It remains unclear whether direct interaction between FVIII and LMAN1 (which has been observed for the cathepsins catC and catZ [70]) is possible, or whether the FVIII–LMAN1 complex forms only with the participation of MCFD2. The specific FVIII motif, which can be recognized by the cargo receptor, has not been identified. This motif is supposed to contain a conformational epitope and a carbohydrate moiety (to ensure that only the correctly folded and post-translationally modified proteins can be transported). The binding motif of LMAN1 has been experimentally identified in the catZ proenzyme molecule; it contains several adjacent N-glycans [37]; however, there are no regions homologous to it in FVIII and FV.

FV and FVIII have similar domain structures, including B domains, which are non-homologous but contain numerous N-glycosylation sites in both cases. Since the B-domain-deleted FVIII is characterized by reduced efficiency in the binding to the LMAN1–MCFD2 complex, a hypothesis has been put forward that LMAN1 predominantly interacts with the B domains [71]. Meanwhile, the blockage of N-glycosylation does not stop the formation of the FVIII–cargo receptor complex [69]; i.e., it is not only the carbohydrate moiety of the molecule, but also the polypeptide chain that participate in the interaction between FVIII and the cargo protein.

The LMAN1–MCFD2 complex specifically recruits FVIII and FV from the ER to COPII (coat protein II) vesicles, which are separated from the ER to subsequently bind to ER GIC (*Fig. 6*). The mechanism of release of the FVIII polypeptide from the LMAN1– MCFD2 complex during its transport has not been elucidated. It is thought to be released due to the change in the local pH value and calcium concentration [71]. The COPII proteins return to the ER outside the vesicles; the ER GIC complexes are subjected to retrograde transport within the COPI vesicles. FVIII seems to be further transported inside vesicles of unknown composition or via transport containers associated with microtubules. FVIII molecules emerge in the Golgi apparatus via the formation of new *cis*-Golgi cisterns [71–73].

## PROCESSING OF FVIII IN THE GOLGI APPARATUS

High-mannose N-glycans of the FVIII molecule are modified in the Golgi apparatus; O-glycosylation and sulfation of tyrosine residues occurs in the *trans*-Golgi. Six active sites of sulfation of tyrosine residues at positions 346, 718, 719, 723, 1664, and 1680 have been detected in human FVIII; they predominantly localize near the acidic subdomains a1, a2, a3 and surround the points where FVIII is cleaved by thrombin. All the six sulfation sites are required to ensure the full activity of factor VIII; the inhibition of sulfation has resulted in a fivefold decrease in the functional activity of FVIII [74]. It has also been demonstrated that sulfation of the Y1680 residue is required to ensure efficient interaction between FVIII and the von Willebrand factor. The natural mutation of Y1680F manifests itself as moderate hemophilia A. In patients carrying this mutation, FVIII retains its normal activity level but is characterized by a decreased half-life value [75]. R.J. Kaufman *et al*. [76] employed site-directed mutagenesis to demonstrate that the presence of sulfated residues at positions 346 and 1664 increases the rate of FVIII activation by thrombin, while sulfation of residues 718, 719, and 723 increases the specific activity of FVIIIa in the tenase complex. The necessity of sulfating residue 1680 so that the complex with the von Willebrand can form has also been confirmed in [76].

The final stage of FVIII processing in the *trans*-Golgi prior to the secretion involves limited proteolysis of the single-chain precursor at residues R1313 and R1648, giving rise to a light and a heavy chain [22]. Both sites of proteolytic processing correspond to the Arg-X-X-Arg motif, which can be cleaved by protease furin/PACE (paired basic amino acid cleavage enzyme); however, it remains unclear what particular signaling protease of the PACE family is responsible for FVIII processing.

## FACTOR VIII IN THE BLOODSTREAM

Mature natural FVIII, which can occur in one of several forms with a molecular weight of 170–280 kDa, is present in the blood plasma at a concentration of 0.1–0.2 μg/ml [77]. Almost all the FVIII in plasma is complexed with a chaperone, the von Willebrand factor, which is secreted by vascular endothelial cells. The FVIII regions responsible for binding to this chaperone have been mapped in the light chain: in the acidic subdomain a3 [78], domains C2 [34, 79] and C1 [80]. The von Willebrand factor stabilizes FVIII in the blood stream and is its key regulator as it allows thrombin to activate the bound FVIII [36, 81, 82] and impedes cleavage of the molecules of nonactivated FVIII by the proteases FXa [83] and activated protein C (APC) [84–86]. Furthermore, vWF prevents the nonspecific binding of FVIII to the membranes of vascular endothelial cells [61] and platelets [87]. It has been demonstrated in *in vitro *experiments that vWF facilitates the association of FVIII chains and the retention of procoagulant activity in the conditioned medium of cells producing FVIII [44, 49]. Similar data have been obtained for re-association of FVIII chains in solution [43, 44]. The dissociation constant of the vWF–FVIII complex is 0.2–0.4 nM; practical equilibrium during the *in vitro *complex formation is attained within seconds [61, 88, 89].

In a significant number of hemophilia A patients, inhibitors of injected exogenous FVIII emerge in the bloodstream, blocking its procoagulation activity [90]. Cases of development of acquired hemophilia A with the normal *FVIII *gene due to the emergence of antibodies against autologous FVIII have also been reported [91]. The etiology of emergence of inhibitory antibodies has not been elucidated; some particular correlations between the emergence of inhibitory antibodies and the HLA haplotype [92] or the nature of the mutation of the factor VIII gene [93] were recently found. IgG antibodies are the predominant class of inhibitory antibodies [94]. Alloantibodies have been found to bind predominantly to the A2 or C2 domains of factor VIII, thus impeding its interaction with factor FIX, whereas autoantibodies are likely to bind to the FVIII C2 domain, which presumably results in blockage of its interaction with phospholipids and vWF [95]. Moreover, it has been demonstrated that anti-factor VIII antibodies can specifically hydrolyze FVIII [96], the proteolytic activity of alloantibodies being in direct proportion to the level of the FVIII inhibitor [97].

## INTERACTION OF FVIII WITH FIXA, FX, AND PHOSPHOLIPIDS

The protein–protein interactions between FVIII (or FVIIIa) and FIXa within tenase are ensured by two different regions; the main contacting surface localizing on the FVIII light chain (*Fig. 1*). The affinity of the free light chain to FIXa is similar to that of the full-length FVIII (*K*_d_ 14–50 nM [67, 98], while *K*_d_ of the full-length FVIII is ~ 2–20 nM [99, 100]). The main site of FVIII –FIXa interaction is a short peptide 1803–1810 [101]; the second site of FVIII–FIXa interaction is the 558– 565 region [67]. The area of direct interaction between FVIII and FX has been found in the acidic C-terminal subdomain of the A1 domain (337–372) [71, 77]; however, this interaction most probably has no significant effect on the function of the tenase complex. The presence of phospholipids is required for FVIII to perform its cofactor function [7, 102, 103]. FVIII interacts *in vivo* with the phospholipids of activated platelets and damaged endothelial cells. Both non-enzymatic cofactors of the coagulation system, FVIII and FV, have been shown to bind to phosphatidylserine [38, 104]. Factor VIII predominantly binds to micelles containing 15–25% of phosphatidylserine, with the dissociation constant reaching 2–4 nM [89, 99, 102]. Platelet activation can increase the phosphatidylserine content in the platelet membrane from 2 to 13%, thus attracting FVIII. FVIII activation increases its affinity to phospholipids by 10 times [105]. The binding site of phospholipids localizes in the FVIII light chain within the C2 domain [106] (*Fig. 5*).

## FVIII ACTIVATION AND FVIIIA INACTIVATION

**Fig. 7:**
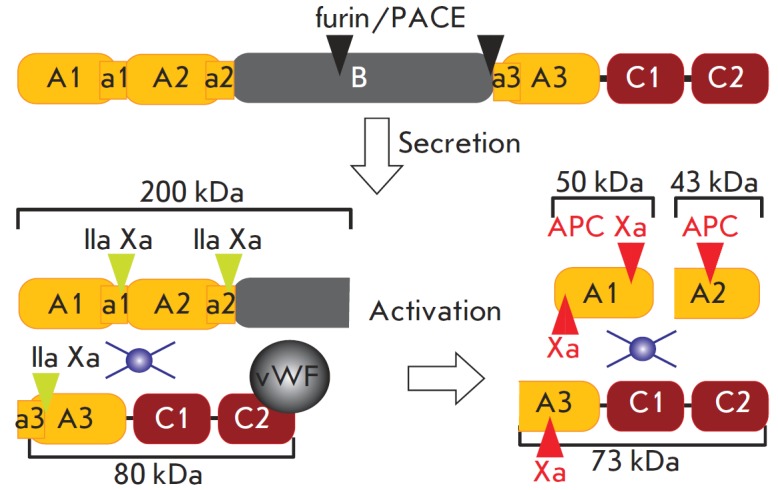
Proteolytic processing of FVIII. Black triangles – sites of processing by the PACE/furin family proteases; green triangles – sites of processing during activation; red triangles – sites of processing during inactivation; and the blue circle represents the coordinated copper ion(s)

*In vivo *activation of FVIII is induced by thrombin or FXa (*Fig. 7*) and involves the introduction of proteolytic breakdowns at several points. When activating FVIII by thrombin, the breakdowns are introduced at positions R372, R740, and R1689 [107] and result in removal of the B domain, in cleavage of the heavy chain into the A1 and A2 domains that remain noncovalently bound, and in elimination of the short acidic region a3 preceding the A3 domain. In a number of publications, the region a3 is referred to as the FVIII activation peptide; however, efficient activation of FVIII cannot be reduced simply to elimination of region a3 from the molecule. FVIII activation by FXa results in cleavage of the FVIII polypeptide chain at the sites specified above and in two or three additional breakdowns at positions R1721, R336, and K36 [63, 107]. Efficient interaction between FVIII and thrombin is mediated by sulfated tyrosine residues in FVIII, while FVIII activation by factor FXa is almost insensitive to the Y → F substitution at sulfation sites [76]. FVIIIa activated by FXa forms tenase that is considerably less productive as compared to that formed by thrombin-activated FVIIIa [108]. Thus, FVIII activation by FXa can be regarded as a side process of FVIIIa inactivation.

FVIIIa inactivation can occur spontaneously and be reduced to the dissociation of the A2 domain of the heavy chain (which is not covalently bound to the remaining FVIII domains) from the FVIIIa molecule [109, 110]. Two specific inactivators of FVIIIa are currently distinguished: APC and FXa. APC cleaves FVIIIa at positions R562 and R336 [71], disintegrating the region of interaction between FVIII and FIX and destabilizing the interaction between the A1 and A2 domains. FXainduced inactivation of FVIIIa seems to occur *in vivo* more rapidly than the APC-induced inactivation does. It involves the introduction of breakdowns at positions R336 and K36 [73], resulting in destabilization of the A1 domain and in the accelerated dissociation of the unbound A2 domain.

## ELIMINATION OF FVIII FROM THE BLOODSTREAM

The FVIII–vWF complex is mainly eliminated from the bloodstream by the specialized clearance receptor LRP (low-density lipoprotein receptor-related protein) that localizes on the hepatocyte membrane [111–113]. A 3.3- fold increase in the half-life of FVIII was observed in* in vivo *experiments in mice when blocking LRP by the receptor-associated 39 kDa protein (RAP) that binds to LRP with high affinity [111]. Three sites take part in the interaction between the FVIII and LRP: the ones in the C2 domain [112], in the A3 domain (1804–1834) [101], and in the А2 domain (484–509) [111]. Multiple sites of FVIII–LRP interaction ensure efficient elimination of unbound chains and the cleaved A2 domain from the bloodstream. The presence of vWF in complex with FVIII within the C2 domain prevents interaction between this domain and LRP, which reduces affinity to LRP by 90% [112]. The *in vivo *interaction of FVIII and its fragments with LRP is mediated by heparin sulfate proteoglycans (HSPG), which interact with the region 558–565 in the A2 domain [114].

## RECOMBINANT FVIII FOR HEMOPHILIA TREATMENT

Pharmaceuticals based on recombinant full-length FVIII were developed almost simultaneously by the biotechnology companies Genetics Institute and Genentech using the *FVIII *gene expression systems in CHO and BHK cells [66, 115] and were approved to be marketed in 1992–1993 with the international nonproprietary name “octocog alfa.” The recombinant FVIII secreted by CHO cells along with the recombinant vWF [49, 77] was produced under the trade names Recombinate^®^ and Bioclate®. The recombinant FVIII secreted by BHK cells into a culture medium containing natural vWF [115] is known under the trade names Kogenate^®^ and Helixate^®^ (*Table 2*).

**Table 2:** Drugs based on recombinant FVIII

Name	Kogenate®, Helixate®	Kogenate FS®, Kogenate Bayer®, Helixate FS®, Helixate NexGen®	Recombinate®, Bioclate®	Advate®	ReFacto®	Xyntha®, ReFacto AF®
Manufacturer	Bayer Healthcare		Baxter		Pfizer	
Generation	1	2	1	3	2	3
Market approvalin USA	1993	2000	1992	2003	2000	2008
Producer cell line	BHK		CHO		CHO	
Heterologous genes	FVIII		FVIII, vWF		FVIII BDD SQ	
Proteins in the culture medium	Human plasma proteins		BSA, aprotinin	-	BSA	-
Immunoaffinity chromatography	+		+		+	-
Stabilizing agent	HSA	Sucrose	HSA	Mannitol, trehalose	Sucrose	
Viral inactivation	SD		Pasteurization	SD	SD	SD, NF

Note. HSA – human serum albumin, BSA – bovine serum albumine, SD – treatment with a solvent and a detergent , NF – nanofiltration.

Today, there are three generations of pharmaceuticals based on recombinant blood-clotting factors [68]: the first-generation drugs contain human serum albumin and contact with animal-derived compounds during the production process; the excipients list of the second-generation drugs contains no albumin; in the third-generation drugs, contact with animal-derived compounds and components of the donated plasma is ruled out during the entire production process. The minimization of the use of plasma components and animal- derived proteins can potentially reduce the risk of transmission of viral and prion infections [116]. No confirmed evidence of transmission of infectious agents when using the first- and second-generation drugs based on recombinant FVIII has been documented thus far.

The production process of full-length recombinant FVIII comprises several stages of ion-exchange chromatography, affinity chromatography using immobilized monoclonal antibodies, and viral inactivation by solvent/detergent treatment or via pasteurization in the presence of a detergent [67, 117]. The recombinant FVIII drugs inevitably contain trace amounts of proteins from the producer cells and murine IgG; thus, the emergence of antibodies against these impurities in patients and the effect of these antibodies on the effectiveness of therapy have been studied during clinical trials. The antibodies formed in most patients; however, the relationship between the immune response to impurity proteins and the effectiveness of therapy has not been elucidated [68].

## B-DOMAIN DELETED RECOMBINANT FVIII

The natural FVIII circulating in the bloodstream contains multiple forms of the truncated B domain, which are formed by proteolysis of a full-length two-chain molecule. The procoagulant properties of these FVIII variants have no significant differences [118]; thus, variants of the recombinant FVIII with targeted deletion of the B domain have been obtained and characterized in a number of studies. The region encoding the amino acid residues 760–1639 (i.e., virtually the entire B domain) was deleted from FVIII cDNA in [118] (*Table 3*).

The procoagulant activity level of FVIII in a conditioned medium for COS-1 cells transfected with a plasmid with cDNA of the deleted FVIII form (LA-VIII variant) was approximately tenfold higher than that of the control cell line transfected with a similar plasmid coding the full-length FVIII. It was ascertained in further studies that the LA-VIII variant is similar to the natural FVIII in terms of its biochemical properties except for the increased sensitivity of the LA-VIII light chain to thrombin cleavage [36]. In more efficient cell lines producing FVIII, B-domain deletion (LA-VIII variant) resulted in a 17-fold increase in the level of FVIII mRN A; however, the concentration of the secreted product increased by only 30% [37]. Similar data were obtained for a FVIII delta II variant, which contained a deletion of the amino acids 771 to 1666 [121]; the level of FVIII secretion in BHK cells reached 0.6 IU/ml, while the secreted product mostly contained the single-stranded 170 kDa form and two additional forms of the heavy chain (120 and 90 kDa) [128]. A similar predominant accumulation of the single-chain FVIII form was also detected in the cases of deletion of regions 741–1648, 741–1668, and 741–1689 [74]. The partial deletion of the amino acid residues 797 to 1562 in the B domain (90-142-80 variant) gave rise to a fully active FVIII [122]; however, this variant when injected into rabbits caused the emergence of specific antibodies against the protein linker region [129], which could potentially have increased the frequency of formation of inhibitory antibodies when used in therapy.

**Table 3:** Deletion variants of FVIII

Sequence	Variant name	Deletion region	Source
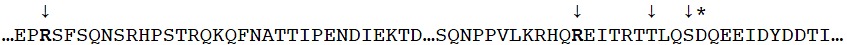	Natural single chain	-	[11]
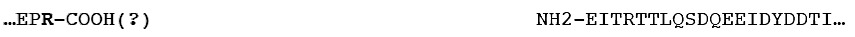	Natural 90+80, seq.1	741?–1648	[119]
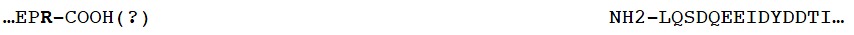	Natural 90+80, seq.2	741?–1654	[119, 120]
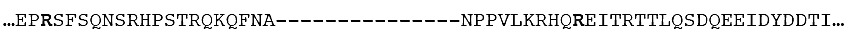	LA-VIII	760–1639	[118]
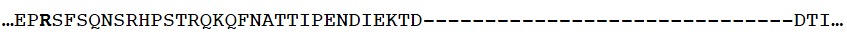	deltaII	771–1666	[121]
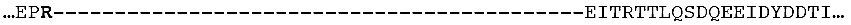	d741-1648	741–1648	[74]
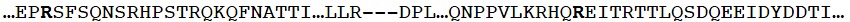	90-142-80	797–1562	[122]
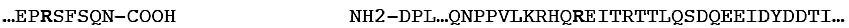	-	746–1562	[123]
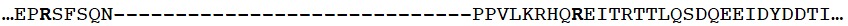	BDD SQ	746–1639	[119]
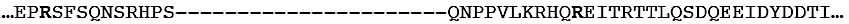	N0, N8	751–1637	[124, 125]
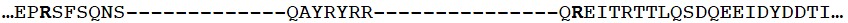	human-cl	747–1640**	[126]
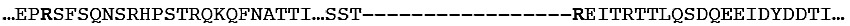	226aa/N6	968–1647	[127]

Note: sign ↓ marks the sites of FVIII processing, cleavage occurs after the amino acid pointed with an arrow;

* – minor processing site of natural FVIII;

** – region 1641–1647 is replaced by an artificial region.

A several-fold increase in the product secretion level as compared to the full-length FVIII has been observed in most heterologous expression systems of B-domaindeleted FVIII. Such change has not been observed for the deletion of the 741–1668 region; however, when the producing CHO cell line was replaced by SK-HEP-1 cells, the secretion level of the deletion FVIII variant increased to 3.5 IU/million cells/day [130]. It is interesting to note that individual expression of the genes coding the truncated heavy and the full-length light FVIII chains with signal peptides of the heavy chain in CHO cells allows one to attain an expression level of 15 IU/ million cells/day [123]. Contamination of the product with the nonprocessed form of the light chain (90 kDa), which seems to carry the C-terminal fragment of the B domain, remains the only (but unavoidable) limitation of this FVIII expression system.

In order to design a recombinant FVIII that would carry neither the B domain nor a non-natural linker region between the heavy and light chains, it is necessary to determine the points of deletion onset and termination, which would make it possible to preserve the availability of the dominant processing sites of natural FVIII for its “minimal” two-chain form (R740 and R1648). When conducting a systematic, exhaustive search, P. Lind *et al*. [119] found that a high level of processing of a single-stranded FVIII precursor at these sites was attained when the amino acid residues 746–1639 were deleted (BDD SQ variant). Meanwhile, no significant proteolytic chain cleavage at other sites has been observed. In this deletion variant, the linking point between the polypeptides of the heavy and light FVIII chains lay in the middle of the separating 14-residue-long linker peptide. The predominant cleavage of the precursor after residues R1648 and S1657 (i.e., the coincidence of the N-terminal light chain region and the natural sequence) was confirmed by Nterminal sequencing of the light chain of the secreted FVIII BDD SQ. The heavy chain of FVIII BDD SQ carried the C-terminal 729–740 region and, partially, the linker peptide [120]. Thus, the BDD SQ variant allows one to reconstruct most accurately the “minimal” twochain FVIII variant that is present in the bloodstream. Variants of the pharmaceutical compositions of the purified FVIII BDD SQ (a solution with a high saccharide content [131] and albumin-free lyophilizate [132]) have been obtained. The lyophilized form of BDD SQ was stable for two years [133] and was used in subsequent clinical trials, which demonstrated its pharmacological effectiveness and safety [134]. Meanwhile, the half-life time of the deletion variant FVIII BDD SQ (international nonproprietary name “moroctocog alfa”) in the bloodstream was slightly lower as compared to the fulllength FVIII obtained from donor plasma.

The original version of the industrial production process of pharmaceutical-grade FVIII BDD SQ (trade name ReFacto^®^) comprised the cultivation of producer cells based on CHO in perfusion bioreactors and the purification of the target protein by five chromatography steps [135]. Viral inactivation was performed by the solvent/detergent treatment of the intermediate product. There are no data on the productivity of the industrially used clonal cell line secreting FVIII BDD SQ. The productivity of a similar cell line based on CHO cells, which had been obtained independently, was 0.5–2.0 IU/ml when grown in a serum-free medium without any induction [136] and up to 10 IU/ml when the product expression was induced by sodium propionate or sodium butyrate. The industrial process of FVIII BDD SQ production was subsequently modified: immunoaffinity chromatography on monoclonal antibodies was replaced by a safer stage comprising affinity purification on an immobilized short peptide [137] (trade names Xyntha^®^, ReFacto AF^®^) (*Table 2*).

The effectiveness and safety of FVIII BDD SQ drugs has been confirmed by clinical trials [138–140]; however, the subsequent meta-analysis of the data of numerous post-marketing studies has cast doubt. R.A. Gruppo* et al*. [141] have demonstrated that prophylactic use of FVIII BDD SQ instead of the full-length FVIII results in a statistically significant increase in the risk of bleeding under prophylaxis. The resistance to slight variations in the initial data (robustness) of the employed meta-analysis method has been discussed in a separate publication and has been confirmed for a wide range of coefficients for recalculating the number of cases of bleeding observed in different studies [142]. The inaccurately measured level of FVIII activity in patients’ plasma as a result of using different coagulometry techniques and standards of procoagulant activity of FVIII [143] could have been one of the reasons for the reduced effectiveness of FVIII BDD SQ in preventive treatment. The reduced half-life time of FVIII BDD SQ [144] as a result of accelerated inactivation of activated FVIII BDD SQ by the activated proteins C and FXa can be presumably considered to be another reason [145].

As for another important safety indicator of FVIII drugs–the risk of emergence of inhibitors–the data for FVIII BDD SQ were rather inconsistent. In some studies, the frequency of emergence of inhibitors was similar for all the variants of recombinant FVIII [146–148], while in other studies an increased risk of inhibitor emergence was observed for FVIII BDD SQ [149]. Since the probability of emergence of inhibitors varies widely depending on the mutation type that caused hemophilia, certain HLA genotypes, and specific features of the substitution therapy, the data obtained in different medical centers may differ to a significant extent [150].

A variant similar to the BDD SQ variant of FVIII with the deleted residues 751–1637 (variant N8, *Table 3*) was obtained via expression in CHO cells [125] and used in clinical trials, which have shown the bioequivalence of N8 to the comparator drug, the full-length recombinant FVIII, after a single administration in a group consisting of 23 individuals [151]. Another industrially applicable gene expression system of the deletion FVIII variant was created using a human HEK293F cell line; the *FVIII *gene contained not the direct deletion of a region in the B domain but a substitution of the 747– 1648 region by the “non-natural” peptide QAYRYRRQ [126] (variant human-cl, *Table 3*). A hypothesis has been put forward that the presence of the processing sites of proteinase Kex2/furin in the artificial linker peptide will allow one to increase the level of processing of the single-chain FVIII. However, the degrees of processing of the single-chain form for BDD SQ and human-cl variants turned out to be almost identical. The expression system of the deletion FVIII variant, which seemed to exhibit the highest efficiency at that moment, was obtained using special hybrid human cells HKB11. The specific productivity of clonal lines for the FVIII variant with the 90-142-80 deletion (*Table 3*) was equal to 5–10 IU/million cells/day [88, 152].

The investigation of the gene expression levels in FVIII variants with the deletion of only the C-terminal fragments of the B domain has demonstrated that the FVIII variant carrying the first 226 amino acids of the B domain and six N-glycosylation sites (variant 226aa/ N6) is secreted by CHO cells fivefold more efficiently as compared with FVIII with the deletion of the fulllength B domain. This is attributable to the improved transport of the precursor protein from the ER to the Golgi apparatus [127] (as compared with the full-length form) and to a decrease in adsorption of the secreted FVIII onto the membrane surface of producer cells [124] (as compared with the regular deletion variants). The productivity of the cells producing the 226/N6 variant based on CHO cells reached 11 IU/ml without inducing target gene expression and 15.7 IU/ml when using a serum-containing medium [52].

## FACTORS LIMITING THE EFFICIENCY OF THE HETEROLOGOUS EXPRESSION SYSTEMS FOR FVIII

A significant decrease in the transcription level of hybrid genes containing an open reading frame (ORF) of* FVIII *were first described in studies devoted to the cultivation of retroviral vectors [153, 154]. The presence of the ORF of *FVIII *did not affect the level of transcription initiation; however, a 1.2 kbp ORF fragment reduced the efficiency of transcription elongation by 30–100 times. The observed effect depended on orientation and, to a significant extent, on the position. It was delocalized as the removal of different parts of the ORF fragment under study resulted in partial recovery of the transcription elongation level. An orientationindependent 305-bp-long transcriptional silencer was later detected in another region of FVIII ORF [155]; its activity was suppressed by sodium butyrate [156], which made it possible to enhance the level of FVIII secretion in the cell culture approximately sixfold. The presence of this controllable element for regulating the transcription level in *FVIII *ORF impedes the production of effective therapeutic viral vectors but has some advantages during FVIII biosynthesis in cell cultures. Stress induced by processing of the FVIII precursor can be limited by suppression of *FVIII *gene transcription in the dividing culture, while only the dense nondividing cell culture is exposed to stress during subsequent induction of *FVIII *expression by adding sodium butyrate.

The codon optimization of the coding region of *FVIII* mRN A was studied in [157]. The replacement of some codons by ones characterized by the highest frequency for *H. sapiens *and the simultaneous elimination of internal TATA-boxes, CHI sites, ribosomal binding sites, cryptic splice sites, etc. from the encoding mRN A region increased the FVIII:C level by 7–30 times. An increase in the ratio between the level of FVIII antigen and its procoagulant activity (the ratio between FVIII:Ag and FVIII:C) from 1.27± 0.3 to 2.35 ± 0.49 was simultaneously observed for the deletion variant FVIII BDD SQ, which can attest to the fact that the practical threshold of productivity of cell line 293 was reached and the nonfunctional protein had appeared in the culture medium.

The FVIII precursor in the lumen of ER forms a stable complex with BiP, the major chaperone [54] and one of the key participants in the UPR signaling pathway. Overexpression of the *FVIII *gene induces transcription of the *BiP *gene [56]; the intracellular level of BiP is proportional to the level of factor FVIII secretion within an appreciably wide range [13, 158]. The suppression of BiP expression by short hairpin RN As (shRN As) increased the secretion level of human FVIII [159] by ~2 times, while the number of copies of *FVIII *mRN A simultaneously decreased by ~65%. A similar effect was observed for the overexpression of the *XBP1 *gene, whose product also participates in UPR.

The overexpression of the chaperone Hsp70 was found to reduce induction of apoptosis in a dense culture of FVIII-producing BHK cells and to increase the level of FVIII secretion [160]. Similar data were obtained for overexpression of the anti-apoptotic genes *Aven *and *E1B-19K *[72]. Meanwhile, no significant changes in the level of *Hsp70 *expression and the anti-apoptotic genes *Bcl-2 *and *Bcl-xL *among clones with different levels of FVIII secretion have been observed for the FVIII-producing human hybrid cell line HKB11 [152]. These data attest to the fact that this pathway of anti-apoptosis re-engineering of FVIII producers can be efficient only for a super-dense BHK cell culture.

The suppression of oxidative stress in the ER (as well as the UPR and apoptosis induced by it) in CHO cells overexpressing FVIII by antioxidants was demonstrated in [51]. The addition of the antioxidant butylated hydroxyanisole to the culture medium simultaneously with sodium butyrate (an agent inducing* FVIII *gene expression) enabled a fourfold increase in the secretion level of the full-length FVIII. Manifestations of oxidative stress were also observed for the FVIII with a fully deleted B-domain but not for the variant 226/N6.

The increase in FVIII secretion by suppressing the intensity of UPR, oxidative stress, and apoptosis of the producer cells may be associated with the changes in the level of FVIII adsorption onto the outer cell membrane. In CHO cells secreting the full-length FVIII, FVIII concentration in a serum-free supernatant increased by a factor of 4 after porcine vWF had been added to the culture medium [77]; i.e., at least three quarters of the total FVIII secreted in the absence of vWF remained bound to the cell membrane. FVIII predominantly binds to phospholipid membranes containing phosphatidylserine. For the membranes of activated platelets, the dissociation constants of FVIII, FVIII BDD SQ, and FVIIIa are equal to 10.4, 5.1, and 1.7 nM, respectively [161]. An increase in FVIII adsorption on the membrane of apoptotic cells (which also contains an elevated fraction of phosphatidylserine) has been shown by flow cytometry. The suppression of apoptosis in producer cells via overexpression of the *Hsp70 *gene resulted in a drop in the level of absorption of the fulllength FVIII on the membrane and an increase in its concentration in the culture medium [161]. In expression systems of the FVIII gene variants with B-domain deletion, adsorption on the cell membrane is more pronounced and can exceed 90% for the variant N0 (which is identical to N8). Partial (instead of complete) deletion of the B domain reduces adsorption to ~ 50%, while the total level of FVIII expression decreases almost twofold [124]. The loss of secreted FVIII N0 on the membrane of the producer cells can also be reduced via the inhibition of its interaction with phosphatidylserine by adding vWF, annexin V, or o-phospho-*L*-serine into the culture medium [162].

## TRANSGENIC ORGANISMS

The expression systems of recombinant proteins of the hemostatic system, which are based on cultured cells, can potentially be completely replaced with technologies that allow production of these proteins in the milk of transgenic animals. Antithrombin III exemplifies the successful implementation of such an approach. For its production, transgenic goats have been developed with antithrombin III productivity of over 1 g/l; industrial processes for purifying the target protein have been elaborated [163]. The level of FIX production in the milk of transgenic pigs was considerably lower [164], which is typically attributed to the insufficient degree of γ-carboxylation of the product. Polypeptide stability and accurate processing of the single-chain form to the heterodimer are considered to be the main factors limiting the productivity of transgenic animals that secrete FVIII in the milk [165]. Functionally active fulllength FVIII was produced in the milk of mice [166], rabbits [167], sheep [168], and pigs [169]; however, in all cases the level of product secretion was of no practical interest (*Table 4*). When the deletion variant FVIII 226/ N6 was used and the von Willebrand factor was coexpressed, the level of FVIII:C in the milk of transgenic mice reached 678 IU/ml, which attests to the possibility of producing large transgenic animals that can secrete industrially significant amounts of FVIII in their milk. However, the simultaneous introduction of two transcriptionally active transgenes into the cattle genome will require a significant effort.

**Table 4:** Main properties of transgenic animals capable of secreting FVIII in milk

Name	Milk volume, l*,**	Estimated maximum productivity, g*,**	FVIII:Ag, μg/ml	FVIII:C, IU/ml	Specific activity, IU/mg, [for plasma 5 000+]	Productivity per doe per year, mg/ IU	Comments & references
Mouse	0.0015	0.01–0.02	50.21	13.41	267	0.075 / 20	Fl [166]
			122–183	555–678	3705–4549	0.183–0.275 / 833–1017	Variant 226/N6 + vWF [165]
Rabbit	2–5	20	0.117***	0.521	4500***	0.234–0.585 / 1042–2605	Fl [167]
Sheep	200–500	2500	N/A	0.02–0.03****	N/A	N/A / 4000– 15000	Fl [168]
Pig	200–400	1500	2.66	0.62	233	532–1064 / 124 000–248 000	Fl [169]

Fl – full-length FVIII.

*Per doe per year.

**According to [170] and [171].

***In the paper cited, the FVIII:Ag content is given in μg/ml, which seems to be a misprint. The data is listed in Table as ng/ml.

****In the paper cited, FVIII:C was measured vs. the standard of natural FVIII and expressed as ng/ml; the data are listed in Table as IU/ml under an assumption that the specific activity of the standard compound is 5,000 IU/mg.

## VARIANTS OF LONG-ACTING FVIII

Despite the fact that the risk of transmission of viral infections has been considerably reduced thanks to drugs based on recombinant FVIII, modern substitution therapy with hemophilia A remains far from perfect, as it denies a decent quality of life to hemophiliacs. The reasons limiting the effectiveness of substitution therapy include the immunogenicity of the injected FVIII, resulting in the appearance of inhibitory antibodies and in FVIII instability in the bloodstream, which requires injections every 2–3 days when FVIII is used as a preventive agent. Since the risk of emergence of anti-FVIII inhibitor antibodies is determined, among other factors, by the number of injections made, production of FVIII derivatives with a prolonged half-life would help to solve both these problems.

The following trends in the study of FVIII derivatives with a prolonged effect can be mentioned: production of FVIII conjugates to hydrophilic polymers, the introduction of point mutations, production of fusion proteins, and design of hybrid human–porcine FVIII variants.

**Fig. 8:**
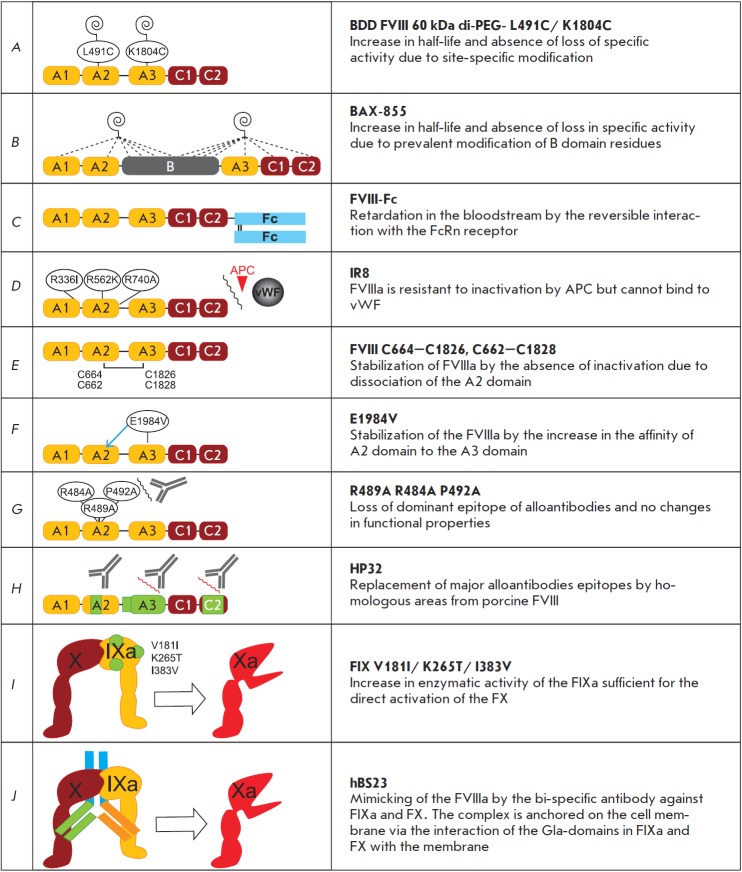
FVIII long-acting variants and functional mimetics. Spirals represent the covalently attached PEG groups; dashed lines – unknown conjugation sites; arrows – noncovalent interaction; and wavy lines – blocking of interactions. Protein parts from porcine FVIII are shown in green in panel H

The conjugation of therapeutic proteins to polyethylene glycol (PEG) molecules usually makes it possible to increase their circulation time in the bloodstream by several times. In some cases, it also reduces immunogenicity and prevents proteolytic degradation. Meanwhile, the non-specific attachment of PEG molecules to the therapeutic protein can cause its inactivation [172]. For FVIII, blockage of its interaction with vWF can also result in a significant decrease in its half-life time. The feasibility of site-specific attachment of PEG molecules to non-paired cysteine residues inserted at various domains of the deletion variant FVIII BDD SQ via sitedirected mutagenesis was studied [173] (*Fig. 8*). For the FVIII variant containing two additional cysteine residues at positions 491 and 1804, which are conjugated to 60 kDa PEG molecules (variant BDD FVIII 60 kDa di- PEG-L491C/K1804C, *Fig. 8A*), an increase in the survival rate of knockout mice after the transection of the tail vein was observed (from 60% for the intact FVIII to 86%) [173]. The non-directed attachment of PEG molecules to the full-length FVIII at lateral amino groups of lysine residues has also allowed to obtain a conjugate (code BAX 855, Fig. 8B) characterized by an average degree of attachment of PEG residues = 2:1, unchanged procoagulant activity, and a lifetime in the bloodstream increased by approximately two times [174].

Since the intact FVIII circulates in the bloodstream within a multimeric high-molecular-weight complex with vWF, there is little promise in increasing the half-life time of the recombinant FVIII by designing proteins fused with long-acting proteins of the blood plasma (e.g., with serum albumin). Meanwhile, linking FVIII in frame to the domains of other proteins, which specifically protect them against elimination from circulation, can considerably increase FVIII stability. Thus, in experiments on knockout mice and dogs with a model of hemophilia A, the protein FVIII–immunoglobulin Fc-region (FVIII-Fc, *Fig. 8C*) provided protection against uncontrollable bleeding twice as longlasting as the intact FVIII [175]. The prolonged effect of FVIII-Fc was entirely determined by the interaction with the neonatal Fc-receptor (FcRn). Clinical trials of FVIII-Fc conducted with 16 patients have demonstrated that the time of retention of FVIII-Rc in the bloodstream (time between the injection of the drug and the decrease in the FVIII:C level below 1%) increases by a factor of 1.53–1.68 [176]. It should be noted that the prophylactic use of FVIII drugs usually includes three injections per week. Meanwhile, the duration of the effect of FVIII needs to be increased at least twice so that a single injection per week is sufficient [177]. Hence, an increase in the duration of the effect of modified FVIII variants by approximately 1.5 times as compared to the intact FVIII can reduce the risk of bleeding to a certain extent for the existing prophylaxis regimens (the socalled “third-day problem”), but it does not allow one to make the injections less frequent.

The alteration of the properties of FVIII via point mutagenesis has been described in several independent studies; however, none of the mutant proteins (muteins) has undergone clinical trials. The introduction of three point substitutions R336I/R562K/R740A to the gene of the deletion variant FVIII 741–1689 (variant IR8,* Fig. 8D*) has enabled the production of a protein characterized by normal procoagulant activity, loss of its affinity to vWF, and high resistance to proteolytic inactivation of FVIIIa by the activated protein C [178]. However, no significant differences in the termination of bleeding in knockout mice have been observed when using this gene variant for targeted FVIII expression on the platelet membrane [179].

The introduction of a pair of cysteine residues to the spatially juxtaposed regions of the A2 and A3 domains forms a disulfide bond between them, which stabilizes the activated FVIII, thus increasing its procoagulant activity [180]. Muteins of the deletion FVIII variant containing the cysteine pair C664–C1826 or C662–C1828 (* Fig. 8D *) in *in vitro *experiments exhibited specific activity tenfold higher than that of the intact FVIII [181].

The stability of FVIIIa can also be enhanced by substituting the amino acids on the interface surfaces between the A2, A1, and A3 domains. The point substitution of E1984V (*Fig. 8F*) resulted in an increase in the lifetime of the activated FVIII by 4–8 times, while its normal procoagulant activity was retained [182].

Most inhibitor antibodies emerging in patients with hemophilia A are oriented toward the epitopes within the A2 and C2 domains. Anti-A2-domain antibod ies mostly interact with a short region, 484–508; thus, the replacement of several amino acids in this region of FVIII can reduce its immunogenicity. It turned out to be sufficient to introduce the triple substitution R484A/ R489A/P492A (*Fig. 8G*) to reduce the average inhibitor level from 670 to 310 Beteshda U/ml in knockout mice that had received seven sequential FVIII injections at an interval of 14 days [183].

It was demonstrated in *in vitro *experiments using a number of hybrid FVIII molecules with the deleted B domain 741–1648, which contained alternating fragments of porcine and human FVIII, that the replacement of the 484–508 fragment of the A2 domain of human FVIII by a homologous fragment of porcine FVIII and complete replacement of the human FVIII A3- and C2 domains by the corresponding domains of porcine FVIII (variant HP32, *Fig. 8H *) allow one to produce a FVIII molecule that is resistant to the inhibitor effect of most antibodies isolated from hemophilia A patients [184]. For this reason, the porcine recombinant FVIII with a deleted B domain (variant OBI-1), which is currently undergoing clinical trials [185], can be potentially replaced by a hybrid molecule that inhibits lower immunogenicity as compared to xenogenic porcine FVIII [186] but carries no immunodominant epitopes of human FVIII.

## FUNCTIONAL ANALOGUES OF FVIII

Since the function of FVIIIa can be confined to increasing FIXa activity, the high-activity analogue of FIXa, which can produce a sufficient amount of thrombin, will ensure efficient blood clotting without the participation of FVIII. Unlike FVIII, FIX modified in this manner can also be used to treat the inhibitor form of hemophilia A. The introduction of a gene therapy plasmid encoding mutein FIX with the triple substitution V181I, K265T, and I383V into *FVIII *gene knockout mice improved the blood clotting indicators [187], thus attesting to the fact that the hemostatic function in patients with hemophilia A can be recovered without using FVIII drugs (*Fig. 8I*). The tenase complex can also be reconstructed by replacing the FVIIIa molecule by a bispecific antibody against FIXa and FX. This antibody was selected among 40,000 molecules composed of fragments of monoclonal antibodies against FIX and FX via high-throughput screening [188]. After the optimization of the structure of the leading molecules, the bispecific humanized antibody hBS23 was obtained, which is capable of increasing the catalytic efficiency of FIXa by 19,800 times (272,000 times for FVIII) due to the 20- fold reduction in *K*_m_ of the reaction of FX activation and a 1,000-fold increase in *k*_cat_ (*Fig. 8J*). A single injection of 0.3 mg/kg hBS23 to macaques used as a model of acquired hemophilia A ensured virtually identical bleeding control as therapy using porcine FVIII.

## CONCLUSIONS

Efficient bleeding control in hemophilia A patients is based on continuous substitution therapy using FVIII preparations and prophylaxis of bleeding in pediatric practice. Since the current state of studies and elaboration of long-acting FVIII derivatives provides no grounds to expect considerably improved drugs in the near future, the development of new cell lines producing FVIII (with allowance for the accumulated knowledge on the FVIII structure and the factors affecting the levels of its biosynthesis and secretion) may enable a several-fold increase in its production. It can be assumed that a simple increase in the production volume of third-generation biosimilar drugs based on recombinant FVIII will make it possible to increase the volume of substitution therapy for hemophilia A, while the current costs remain unchanged (i.e., to improve patients’ quality of life and increase their longevity without reallocating the limited healthcare resources).

## Abbreviations

FVIII: blood clotting factor VIII
BDD: B domain deleted
IU: international unit;
FVIII:Ag: concentration of FVIII antigen
FVIII:C: procoagulant activity of FVIII
vWF: von Willebrand factor. Addition of “a” to the number of the corresponding clotting factor denotes the activated factor
